# Typologies of stress appraisal and problem-focused coping: associations with compliance with public health recommendations during the COVID-19 pandemic

**DOI:** 10.1186/s12889-022-13161-5

**Published:** 2022-04-19

**Authors:** Justin F. Landy, Aya Shigeto, Daniel J. Laxman, Lawrence M. Scheier

**Affiliations:** 1 Department of Psychology and Neuroscience, College of Psychology, Nova Southeastern University, 3301 College Avenue, FL 33314 Fort Lauderdale, USA; 2 University Office of Evaluation and Educational Effectiveness, Arizona State University, Tempe, AZ USA; 3 LARS Research Institute, Inc, Scottsdale, AZ USA

**Keywords:** Coping, COVID-19, Latent class analysis, Public health, Stress

## Abstract

**Background:**

Given prior research finding that young adults are less likely to engage in recommended public health behaviors (PHBs) than older adults, understanding who is and is not likely to engage in PHBs among young adults is crucial to mitigating the effects of the COVID-19 pandemic. Drawing on the Transactional Theory of Stress and Coping, this study examined how typologies of stress appraisal (SA) and problem-focused coping (PFC) among young adults were associated with compliance with public health recommendations during the pandemic.

**Methods:**

An online sample of young adults in the United States, ages 18–35, was recruited during the early phase of the pandemic (April–May 2020). Participants reported their appraisals of how central, threatening, and uncontrollable the pandemic was, their tendencies to engage in instrumental, problem-focused coping strategies, and how frequently they engaged in three recommended PHBs (social distancing, mask wearing, and hand washing).

**Results:**

Using latent class analysis, we identified three classes of individuals: Low-SA/Low-PFC, Low-SA/High-PFC, and High-SA/High-PFC. Demographics did not efficiently distinguish membership in the three classes. The former two classes reported less compliance with public health recommendations than did the latter class. Tests of measurement invariance for gender indicated trivial differences in the composition of class membership and relations to compliance.

**Conclusions:**

This research uncovered three qualitatively distinct classes of people who differed in their appraisal of the pandemic and their tendency to engage in PFC. Individuals who view the pandemic as central and threatening and engage in problem-focused coping were more likely than their peers to comply with guidelines recommending social distancing, mask wearing, and hand washing. These results contribute to our understanding of why people do and do not comply with public health guidelines and highlight the importance of attending to psychological variables in public health research. Understanding what drives poor compliance with public health recommendations can contribute to efforts promoting better compliance, and ultimately better health outcomes.

## Background

The novel coronavirus disease 2019 (COVID-19) pandemic constitutes a public health crisis on a scale unseen since the 1918 influenza pandemic. As of mid-March, 2022, there were over 458 million confirmed cases of COVID-19 worldwide, including over 6 million deaths [1]. In the United States alone, over 79 million cases and 965,000 pandemic-related deaths have been documented [2].

The toll of the pandemic has not just been physical, but also psychological, as people have dealt with the pandemic and its many consequences, including economic repercussions [3–5], negative effects of social distancing and quarantine on mental health [6–8], fear that oneself or one’s loved ones might contract the virus [9–11], and, of course, grief from losing loved ones to the pandemic [12–14]. Fortunately, even before the recent development of safe and effective vaccines, research clearly showed that the spread of the virus could be mitigated by engaging in evidence-based public health behaviors (henceforth, PHBs), particularly washing hands, wearing masks, and social distancing [15]. These behaviors are among several recommendations from the CDC [16] and WHO [17] to suppress the spread of the virus. However, academic research [18, 19], as well as polls by Harris and Gallup [20, 21], have shown high but not universal levels of compliance with these guidelines throughout the pandemic. In particular, some studies indicate that younger adults are less likely to engage in PHBs than older adults [22, 23]. Therefore, understanding who is and is not likely to engage in PHBs among young adults is crucial to mitigating the effects of the virus. Given the stressful nature of the pandemic and the importance of engaging in PHBs, this study examined the relations between stress appraisal of the pandemic, problem-focused coping, and compliance with public health recommendations among young adults.

### Stress appraisal and problem-focused coping

The most common theoretical framework for understanding an individual’s experience of stress and their choice of how to respond is Lazarus and Folkman’s Transactional Theory of Stress and Coping (TTSC) [24–26]. TTSC is a cognitive-relational theory of how an individual interacts with their environment when faced with a stressor. The interaction is dynamic and bidirectional, given that a person’s response to the stressor changes as the nature of the stressor, and thus the person’s appraisal of it, changes. The theory posits that stress elicits a coping response through stress appraisal (henceforth, SA), with the latter involving two cognitive processes, primary and secondary appraisal. When faced with a potential stressor, individuals engage in *primary appraisal* whereby they evaluate the potential stressor in terms of its characteristics, such as *centrality, uncontrollability,* and *threat*. A stressor (e.g., a stimulus or event) is considered central if it is perceived to have direct, negative consequences to the self, uncontrollable if it is perceived as being outside of one’s own control, and threatening if it is perceived as anxiety-inducing and having a negative outcome. These three aspects of stress appraisal are in keeping with TTSC’s original themes of harm/loss, threat, or challenge [26]. Collectively, these different components of SA help to shape the personal meaning of stress and regulate the impact of stress on psychosocial functioning. Folkman further articulated that different dimensions of primary appraisal need not necessarily co-occur, and people can show complex patterns in how they appraise stress (e.g., viewing the stressor as being central and threatening, but not uncontrollable) [24].

According to TTSC, if people determine a stimulus or event to be something that they need to respond to, then the primary appraisal is followed by a *secondary appraisal* in which they evaluate whether their actions can ameliorate the impact of the stressor by considering their coping strategies (e.g., problem-focused coping, emotion-focused coping) and coping resources (e.g., physical, social, psychological, and material assets). Coping strategies are responses aimed at mitigating stress and are typically categorized into two types: problem-focused and emotion-focused coping. *Problem-focused coping* (henceforth, PFC) consists of active efforts to instrumentally manage the problem that is causing stress. *Emotion-focused coping* is a reactive effort to remove the negative emotional state itself without addressing its cause, for instance by venting, dismissing, or diverting one’s attention. In the context of the pandemic as a stressor, PHBs such as washing hands, wearing masks, and social distancing are direct, active strategies that individuals can use to prevent the spread of the virus and protect themselves and others from infection. In other words, they are strategies that are aimed at mitigating the stressor itself, rather than attenuating negative emotional states. Therefore, PFC, but not emotion-focused coping, is expected to be particularly relevant to people’s compliance with public health recommendations.

Individuals may differ from one another not only quantitatively (i.e., mean differences) in terms of their appraisal of stressors and use of coping strategies but also qualitatively (i.e., patterns of stress appraisal and use of coping strategies). Conceivably, individuals who exhibit similar patterns can be classified into unique subgroups. Such an approach involves examining subgroup heterogeneity (i.e., the potential existence of unique subgroups with distinct response patterns) using a person-centered approach [27]. Although prior research has examined individual differences in SA and coping strategies, we are not aware of any research that has specifically addressed the question of whether there are “typologies” of people who exhibit unique patterns of both SA and coping strategies. Several researchers [25, 28] have alluded to this type of subgroup heterogeneity; however, it has not been rigorously examined using any form of cluster-based analytic technique.

Another important area of concern is how SA and coping strategies relate to compliance with recommended PHBs. There is some empirical evidence suggesting that those who consider the coronavirus more severe and more controllable are more likely to engage in PHBs [29–31]. Additionally, some research has found a link between active coping approaches and positive attitudes and skills regarding COVID-19 prevention and protection [32]. However, to our knowledge, no research has examined primary appraisals and coping strategies *simultaneously* to identify typologies and then related them to PHBs. The spate of recent studies that examined compliance with public health recommendations has generally not done so within a stress-coping framework [18, 33, 34]. Moreover, to our knowledge, only one study on this subject used a person-centered approach to study PHBs during the pandemic [29], but the classification strategy was applied to PHBs, not stress-coping.

### The present study

In the present study, we used latent class analysis (LCA) to examine subgroup heterogeneity (that is, typologies or “classes”) based on SA and PFC. By examining how individuals both appraise and respond to a stressor (i.e., the pandemic), the present research directly captures the transactional nature of coping. Folkman and Lazarus considered their theory “transactional” because people use information from their appraisal of a stressor to modify their coping responses as they “transact” with the stressor. Conversely, individuals can modify their appraisal of the stressor based on the coping response they use.

The use of a person-centered approach like LCA differs from traditional variable-centered approaches because it does not examine relationships among variables that are often assumed to apply to all people. Rather, LCA focuses on an individual’s set of responses as a whole and classifies them into subtypes based on their item endorsement patterns (e.g., likelihood of engaging in a specific PFC behavioral response) [35]. High consistency in item endorsement patterns is used to characterize the unique composition of classes. Once membership in the mutually exclusive classes has been determined, models can be covariate-adjusted to learn more about the distinctive characteristics associated with class membership. In the current study, we examined age, gender, race, employment status during the pandemic, living situation, education, and sample recruitment strategy. Latent class membership can also be used to predict an observed distal outcome, which, in the current study, is compliance with public health recommendations during the early phase of the pandemic. Moreover, given increasing empirical evidence that women are more likely than men to engage in PHBs during epidemics and pandemics [29, 36–39] as well as in health behaviors more generally [40], we examined possible gender differences in the relations between typologies of stress appraisal and problem-focused coping and PHBs.

## Method

### Participants

Participants were recruited using two methods: Amazon’s Mechanical Turk (MTurk) labor pool (i.e., an online platform where participants can complete simple tasks such as surveys in exchange for monetary compensation) and snowball sampling through University colleagues and research assistants. Regardless of recruitment method, all participants were screened to ensure that they were between the ages of 18 and 35 (details on the recruitment and screening procedures are presented in [41]). The project was approved by the University Institutional Review Board (IRB) at the first author’s institution, and carried out in accordance with the ethical guidelines of the American Psychological Association.

A total of 1,509 individuals initiated the survey on the Qualtrics platform. From this sample a total of 154 were eliminated, including 87 respondents (5.77%) who failed at least one attention check [42], and 67 respondents (4.44%) who did not provide answers to any of the 12 items of SA, any of the 7 items of PFC, or the 3 items of PHBs, producing a final sample of *N* = 1,355. The sample was fairly young, as expected (*M*_*Age*_ = 26.67, *SD* = 4.76), and racially and ethnically diverse (64.58% White/European American, 13.14% Asian, 6.94% Latino/Latina/Latinx/Hispanic, 6.72% Black/African American, 0.52% American Indian or Alaska Native, 7.31% Multiple racial/ethnic identities, 0.81% Other). Less than half the sample was employed (42.88%). Most participants were either attending college (32.99%) or had an undergraduate degree (34.32%), with smaller portions having less education than a college degree (19.26%) or a graduate degree (13.34%). Most lived with family members (79.19%), with smaller numbers living alone (13.28%) or with non-family roommates (7.53%). Most participants (83.69%) were recruited via MTurk, with the remainder recruited via snowball sampling. We did not collect information on where participants lived within the United States.

### Survey procedure

The survey recruitment phase ran for three weeks from Tuesday, April 14, 2020, to Tuesday, May 5, 2020, coinciding with the early stages of the pandemic. Both MTurk and snowball method participants accessed the Qualtrics online survey platform and, after providing informed consent, responded to a series of demographics questions. Following this procedure, participants were presented with the main set of questions assessing stress appraisal and coping along with psychosocial measures. The survey employed a randomized three-form planned missingness design [43, 44] to keep the length of the survey manageable. Each participant responded to either 71 or 73 items, approximately two-thirds of the total number of items (for more detail, see [41]).

### Measures

#### Stress appraisal

We measured SA using 12 items from the centrality, uncontrollability, and threat subscales of the Stress Appraisal Measure [45]. Four items from each subscale were modified to refer specifically to the COVID-19 pandemic, as we were interested specifically in primary appraisals of this particular stressor. Participants rated their agreement with each item on a 5-point scale (1 = *Not at all*; 2 = *Slightly*; 3 = *Moderately*; 4 = *Considerably*; 5 = *Extremely*). Internal consistencies for the centrality, uncontrollability, and threat subscales using McDonald’s Omega [46] were ω = 0.86, ω = 0.71, and ω = 0.74, respectively. We dichotomized responses for LCA with responses of 1–3 coded as “0” and responses of 4–5 coded as “1.” Importantly, the usual objections to dichotomizing continuous variables (e.g., [47, 48]) do not apply in a person-centered analytic framework. This is because the pattern of responses derives no information from the underlying variance of a measure but rather from the probability of survey respondents endorsing a particular response. In this regard, LCA uses a contingency table for analysis as opposed to a covariance structure.

#### Problem-focused coping

We measured PFC using seven items from the Coping Assessment Battery [49, 50]. Participants indicated how often they employed each coping strategy (e.g., “Think about which of the alternatives is best”) on a 5-point scale (1 = *Never*; 2 = *Rarely*; 3 = *Sometimes*; 4 = *Often*; 5 = *Almost always/always*). Internal consistency was ω = 0.79. For LCA, we coded responses of 1–3 as “0” and 4–5 as “1.”

#### Compliance with public health recommendations

Participants indicated how often they engaged in three PHBs (i.e., social distancing, mask wearing, and hand washing) on a 5-point scale (1 = *Not at all*; 2 = Rarely; 3 = *Sometimes*; 4 = *Often*; 5 = *Almost always/always*). For analytical purposes, we treated the three items as a unit-weighted risk index assessing poor compliance, coding responses of 1–3 as “high-risk (1)” and 4–5 as “low-risk (0)” for each item. The score (a count of poor compliance with the three PHBs) ranged from 0 to 3, with a higher score representing higher risk (i.e., lower compliance).

#### Demographics

Demographic measures were coded as follows: gender (woman = 0, man = 1), race (non-White, including Latino/Latina/Latinx/Hispanic = 0, White = 1), employment (not employed = 0, employed = 1), residential status–with family (living alone = 0, living with family = 1), residential status–with roommate(s) (living alone = 0, living with a non-family roommate(s) = 1), education–earned degree (being in college = 0, having a postsecondary degree = 1), education–some schooling (being in college = 0, having limited or no postsecondary education = 1), and recruitment method (recruited via MTurk = 0, or via snowball method = 1).

#### Missing data treatment

Missing data estimation for the planned missingness design was handled using R version 4.0.0 [51] and RStudio version 1.3.1073 [52] with the Multiple Imputation by Chained Equations (MICE) procedure [53] (see [41] for details of imputation for this study). This method has been shown to produce efficient parameter estimates and unbiased standard errors [54] and is superior to ad hoc methods such as listwise deletion [43]. The complete-data analyses are based on 20 imputed data sets conducted with the Mplus statistical package [55]. Model estimates are based on averaging the 20 imputations accounting for missing data uncertainty.

#### Model testing strategy

Model extraction proceeded from a 2-class model to an 8-class model using the 19 indicators (i.e., 12 SA items and seven PFC items). Several inferential statistics were used to select the best-fitting model, including the Akaike Information Criteria [56], Bayesian Information Criteria [57], changes in the Log Likelihood (L^2^) statistical fit index with the addition of new classes [58], and entropy. Entropy provides an estimate of classification “uncertainty” or chaos in the model and is based on the estimated posterior probabilities [59]. Moreover, the class structure should be interpretable and show clear separation between classes. The latter criterion requires inspecting the item response probabilities (i.e., the likelihood that members of a class endorsed an item) to determine whether they clearly distinguish uniquely identifiable and qualitatively discrete classes [35, 60]. Composition of class membership was based on a 0.60 cut-off for the item response probabilities (i.e., members of a class had > 0.60 likelihood of endorsing a particular item). We did not consider classes with very small samples to avoid sparse cells and convergence problems that can arise from weak identifiability [61].

As a second step in the modeling process, we examined the influence of covariates on class membership using multinomial logistic regression (MLR). This procedure determines whether there are distinct individual characteristics uniquely associated with class membership. We used the R3STEP utility available in Mplus [62, 63] to test the covariate-adjusted models. This procedure prevents the measurement parameters that help define class membership from being influenced by covariates, which should be structurally independent of the class measurement model.

We next modeled the effect of class membership on compliance with public health recommendations. The unit-weighted risk index (i.e., poor compliance) was modeled as an observed “distal” outcome allowing intercepts to vary across classes. The index was treated as a count; that is, following a Poisson distribution. Specifically, for each class, the mean of the log risk index score was estimated and covariate adjusted using any significant covariates, resulting in a covariate-adjusted intercept for each class. The intercepts were exponentiated to obtain a covariate-adjusted count of poor compliance across the three PHBs. The count for each class was then compared using pairwise comparisons to determine whether classes differed in their risk of poor compliance. A positive difference in mean covariate-adjusted counts across the classes would indicate that members of a particular class were at greater risk of poor compliance than members of another class.

A final step involved estimating separate models based on gender. This multiple group procedure includes establishing configural invariance (the same number of classes), metric invariance (setting the threshold parameters to equivalence across gender groups), and equivalence of latent class prevalence. The multiple group model was then tested in the same manner as the full sample model, first covariate-adjusting the model and then predicting the risk index from class membership, separately for male and female participants. A Monte Carlo simulation (100 replications) using hypothesized population parameters indicated adequate coverage of the parameter estimates (> 95%), negligible standard error bias (< 5%) and power exceeding 0.95 for thresholds with a 3-class covariate-adjusted model [64].

## Results

Response frequencies for each of the three public health behaviors (PHBs) were as follows: 16 (1.18%) engaged in *social distancing “*not at all”, 19 (1.40%) “rarely,” 70 (5.17%) “sometimes,” 292 (21.55%) “often,” and 958 (70.70%) “always/almost always”; 251 (18.52%) engaged in *mask wearing* “not at all,” 163 (12.03%) “rarely,” 260 (19.19%) “sometimes,” 338 (24.94%) “often,” and 343 (25.31%) “always/almost always”; 8 (0.59%) engaged in *hand washing* “not at all,” 11 (0.81%) “rarely,” 68 (5.02%) “sometimes,” 457 (33.73%) “often,” and 811 (59.85%) always/almost always. For the risk (or poor compliance) index based on dichotomization of each of the PHBs (i.e., 0 for responses of 4 [Often] and 5 [Always/almost always]; 1 for responses of 1 [Not at all], 2 [Rarely], and 3 [Sometimes]), 644 participants (47.53% of the sample) had no risk, reflecting compliance with all three PHBs, 576 (42.51%) had 1 risk factor, reflecting compliance with two PHBs, 115 (8.49%) had 2 risk factors, indicating compliance for only one PHB, and 20 (1.48%) endorsed 3 risk factors, indicating compliance with none of the PHBs.

### Latent class analysis

The top portion of Table 1 shows fit indices for the LCA model testing sequence based on the entire sample. With increasing extraction of classes, there was a corresponding decrease in the AIC and BIC values, with a concomitant increase in entropy reaching its largest value with the 3-class model. Inspection of models with additional classes indicated less than optimal fit and poor latent class enumeration with single items identifying class membership. Table 2 shows the conditional item response probabilities for the 3-class model. Members of Class 1 (“Low-SA/Low-PFC”; 22.26%) did not endorse any items above the 0.60 critical threshold. Members of Class 2 (“Low-SA/High-PFC”; 39.52%) did not endorse any of the SA items but did endorse the PFC items, with one exception (PFC7; “Compromise to get something positive from the situation”). Members of Class 3 (“High-SA/High-PFC”; 38.22%) endorsed the SA-Centrality and SA-Threat items with one exception (SA-Threat4; “This is going to have a negative impact on me”), and the 7 PFC items with the exception of the same compromise item as above (PFC7).

**Table 1:** Model Fit Statistics for Latent Class Analyses: Stress Appraisal & Problem-Focused Coping

Classes	LL (Deviance)	No. of Parameters	AIC	BIC	Relative Entropy
Entire sample (*N* = 1,355)					
2	-14,643.168	39	29,364.34	29,567.59	.804
**3**	**-14,173.596**	**59**	**28,465.19**	**28,772.67**	**.815**
4	-14,020.537	79	28,199.07	28,610.79	.801
5	-13,889.167	99	27,976.33	28,492.29	.779
6	-13,789.066	119	27,816.13	28,436.31	.774
7	-13,724.403	139	27,726.81	28,451.21	.793
8	-13,685.043	159	27,688.09	28,516.72	.801
Women only (*n* = 821)					
2	-8834.239	39	17,746.48	17,930.19	.801
3	-8569.333	59	17,256.66	17,534.59	.814
4	-8476.134	79	17,110.27	17,482.39	.803
5	-8391.995	99	16,981.99	17,448.33	.792
6	-8322.052	119	16,882.11	17,442.66	.784
7	-8271.293	139	16,820.59	17,475.35	.798
8	-8244.528	159	16,807.06	17,556.03	.805
Men only (*n* = 534)					
2	-5754.013	39	11,586.03	11,752.96	.819
3	-5532.094	59	11,182.19	11,434.73	.833
4	-5461.489	79	11,080.98	11,419.13	.837
5	-5392.192	99	10,982.38	11,406.14	.826
6	-5352.988	119	10,943.98	11,453.34	.839
7	-5339.384	139	10,956.77	11,551.74	.840
8	-5311.558	159	10,941.12	11,621.70	.848

*LL* Log-likelihood statistics, *AIC* Akaike Information Criterion, *BIC* Bayesian Information Criterion

Model fit indices reflect mean values over 20 imputations adjusted for uncertainty. Relative entropy is a summary measure of classification certainty once posterior class probabilities are obtained and can be computed for k > 1-class models. Some model fit statistics (e.g., Lo-Mendell-Rubin likelihood ratio test) are not available with multiply imputed data

**Table 2:** Item Response Probabilities for the 3-Class Model

	Latent Class		
	1	2	3
	Low SA/ Low PFC	Low SA/ High PFC	High SA/ High PFC
Prevalence	22.26%	39.52%	38.22%
SA-Centrality1	0.265	0.161	**0.789**
SA-Centrality2	0.304	0.156	**0.856**
SA-Centrality3	0.226	0.060	**0.783**
SA-Centrality4	0.238	0.112	**0.732**
SA- Uncontrollability1	0.182	0.038	0.194
SA- Uncontrollability2	0.324	0.184	0.355
SA- Uncontrollability3	0.217	0.109	0.218
SA- Uncontrollability4	0.198	0.097	0.244
SA-Threat1	0.331	0.241	**0.705**
SA-Threat2	0.324	0.307	**0.605**
SA-Threat3	0.382	0.463	**0.709**
SA-Threat4	0.255	0.183	0.491
PFC1	0.316	**0.940**	**0.871**
PFC2	0.302	**0.885**	**0.805**
PFC3	0.377	**0.880**	**0.840**
PFC4	0.254	**0.922**	**0.891**
PFC5	0.210	**0.756**	**0.822**
PFC6	0.389	**0.753**	**0.775**
PFC7	0.270	0.547	0.493

*SA* Stress Appraisal, *PFC* Problem-Focused Coping

The bolded item response probabilities represent the threshold of .60 or higher

### Association between covariates and class membership

The top portion of Table 3 shows the results of the covariate-adjusted models, with each covariate entered into the model individually. The bottom portion of the table shows the results when all covariates are entered simultaneously. Class 1 (Low-SA/Low-PFC) was considered the reference class because members generally do not endorse any PFC strategies. In the fully adjusted model, men were less likely to be members of Class 3 (High-SA/High-PFC) compared to the reference class (OR = 0.582, *p* < 0.05, 95% CI [0.385, 0.882]). Additionally, participants who were recruited via the snowball method were more likely to be members of Class 2 (Low-SA/High-PFC) compared to the reference class (OR = 2.298, *p* < 0.05, 95% CI [1.216, 4.343]).

**Table 3:** Results of Multinomial Logistic Regression Predicting Class Membership

	Latent Class		
	1	2	3
	Low SA/ Low PFC	Low SA/ High PFC	High SA/ High PFC
Prevalence	22.26%	39.52%	38.22%
Unadjusted Odds Ratio			
Age^a^	Ref	1.046^*^	1.010
Gender^b^	Ref	0.927	0.594^**^
Race^c^	Ref	1.114	1.231
Employment^d^	Ref	1.589^*^	0.953
Living with Family^e^	Ref	1.141	0.741
Living with Roommate^f^	Ref	0.865	1.324
Education – Earned Degree^g^	Ref	1.450^†^	1.374
Education – Some Schooling^h^	Ref	1.053	0.667
Recruitment Method^i^	Ref	1.626	1.689^†^
Adjusted Odds Ratio			
Age	Ref	1.037	1.016
Gender	Ref	0.914	0.582^*^
Race	Ref	1.062	1.220
Employment	Ref	1.457^†^	0.874
Living with Family	Ref	1.197	0.677
Living with Roommate	Ref	1.039	1.002
Education – Earned Degree	Ref	1.441	1.339
Education – Some Schooling	Ref	1.411	0.788
Recruitment Method	Ref	2.298^*^	1.824^†^

*Ref* Reference class

^a^Continuous variable

^b^Woman = 0, Man = 1

^c^Non-White (including Latino/Latina/Latinx/Hispanic) = 0, White = 1

^d^Not employed = 0, employed = 1

^e^Living alone = 0, living with family = 1

^f^Living alone = 0, living with non-family roommate(s) = 1

^g^Being in college = 0, having a postsecondary degree = 1

^h^Being in college = 0, having limited or no postsecondary education = 1

^i^Being recruited through MTurk = 0, being recruited through snowball method = 1

Assignment to class is based on the most likely latent class membership, using the latent class posterior distribution

^†^*p* < .10, ^*^p < .05, ^**^p < .01, ^***^p < .001

### Distal outcome

Poor compliance with public health recommendations was predicted from class membership as described in the method section. The count of poor compliance across the three PHBs was covariate-adjusted using only significant covariates (i.e., gender and recruitment method). The covariate-adjusted count of poor compliance across the three PHBs was compared across the three classes using pairwise comparisons to determine whether the classes differed in terms of the level of poor compliance. Results showed that members of Class 1 (Low-SA/Low-PFC) were at significantly greater risk of poor compliance than members of Class 3 (High-SA/High-PFC; mean difference in covariate-adjusted count = 0.763 [Class 1]—0.512 [Class 3] = 0.251, *p* < 0.001). Members of Class 2 (Low-SA/High-PFC), compared to Class 3 (High-SA/High-PFC), were also at significantly greater risk of poor compliance than members of Class 3 (mean difference in covariate-adjusted count = 0.695 [Class 2]—0.512 [Class 3] = 0.183, *p* < 0.001).

### Multiple group model

We next tested for measurement invariance between male and female participants by constraining the thresholds between the same classes (see Table 1). Entropy was good for this model (0.889), and a nested comparison of the unrestricted model with one restricting thresholds to equivalence across the two groups was significant, ΔL^2^(57 free parameters) = 139.806, *p* < 0.001, indicating that the constraint on the between-group thresholds was not tenable. A second nested comparison for the latent class prevalences indicated that the probabilities of being assigned to the various classes were not significantly different for men and women, ΔL^2^(2 free parameters) = 4.544. Pairwise comparisons of PHB compliance within the same classes (e.g., women in Class 1 vs. men in Class 1) indicated no significant differences, although women did report directionally lower risk of poor compliance in each class than men.

## Discussion

This study identified unique typologies of stress appraisal (SA) and problem-focused coping (PFC) in the context of the early stages of the COVID-19 pandemic and examined how they relate to compliance with public health recommendations (i.e., social distancing, mask wearing, and hand washing). SA and PFC were conceptualized as dynamically intertwined cognitive-behavioral processes reflecting underlying typologies that characterize how people perceive and deal with stress. The different typologies reflect unique item endorsement patterns capturing different ways people cope with and respond to stress. The notion of typologies has rarely been tested in the context of the Transactional Theory of Stress and Coping (TTSC), with one exception [41]. In the current study, measures of SA were specific to the pandemic, and measures of PFC reflected instrumental strategies that people apply to diminish or eliminate stress. Typical PFC strategies include thinking about information to solve problems, deliberating choices, getting information, weighing alternatives, and considering risks and consequences. Then, we estimated the mean risk for poor compliance and compared that risk across the three typologies, which has important ramifications for public health initiatives.

The results of the LCA supported a three-class model. The classes were distinguished based on the extent to which participants appraised the pandemic as being central to their lives (e.g., having important consequences and serious implications) and threatening (e.g., making the person anxious, feeling it will have a negative impact) and the extent to which they implemented instrumental coping strategies to mitigate stress. Interestingly, the perceived uncontrollability of the pandemic did not efficiently distinguish classes and was not highly endorsed in any class. The largest class was characterized by participants who did not perceive the pandemic as particularly stressful and endorsed using PFC strategies. Conversely, the smallest class was characterized by individuals who neither perceived the pandemic as especially stressful nor routinely applied PFC strategies. Interestingly, very few of the examined covariates helped to distinguish class membership. Only female gender was associated with membership in Class 3 (High-SA/High-PFC) compared to the reference class (Class 1, i.e., Low-SA/Low-PFC). Having been recruited via the snowball method was associated with membership in Class 2 (Low-SA/High-PFC) compared to the reference class.

Comparison of mean levels of poor compliance with public health recommendations between classes showed that members of Class 1 (Low-SA/Low-PFC) and Class 2 (Low-SA/High-PFC) reported less compliance than members of Class 3 (High-SA/High-PFC). This indicates that compliance was higher among individuals who view the pandemic as central and threatening (but not uncontrollable) and also possess a tendency to cope with stressors using an active, problem-solving approach. Members of Class 2 (Low-SA/High-PFC) also possess this tendency, but do not perceive the pandemic as particularly stressful. The unique differences between classes illustrate the interactive process of stress-coping: individuals who appraise the pandemic as particularly stressful *and* regularly engage in PFC strategies are more likely to comply with public health recommendations than those who do not share the same appraisal of the pandemic and/or do not have PFC in their coping repertoire. Conceivably, the stress induced by the pandemic encourages these individuals to apply their preexisting PFC skills, which then motivates them to engage in recommended public health behaviors (PHBs), as this represents the best strategy for minimizing the effects of the virus.

Gender analyses did not reveal any substantive differences in the composition of classes. Females reported lower levels of risk for poor compliance compared to males, but this difference was not statistically significant. Although lack of gender difference in PHBs has been reported in India [31], the finding of few gender differences in compliance is not fully consistent with past research [29, 36–40]. However, it is important to note that the meta-analysis conducted by Moran and Del Valle [39] was based on studies that used variable-centered approaches with an emphasis on mean gender differences. In the current study, gender differences were based on unique response patterns, rather than mean differences. Additionally, other studies that found higher compliance with PHBs among women included a wide range of ages from 18 to late adulthood (age 65 and above) [29, 36, 37] and were all conducted outside the US [29, 36–38]. More studies with a person-focused approach including LCA as well as with consideration of cultures are needed to explore potential gender differences, or lack thereof, in PHBs.

### Limitations and future directions

There are several limitations of this study worth noting. The method of participant recruitment may have led to some bias in the model estimates. This is because we used both a crowdsourcing labor pool (i.e., MTurk) and a snowball recruitment method to increase the sample size. Importantly, recent evidence suggests that there is minimal bias associated with obtaining data from crowdsourcing web services like MTurk [65, 66]. Moreover, to ensure the validity of responses, we included instructional manipulation checks assessing attentiveness to instructions [42, 67]. There is evidence showing that attention checks result in superior performance by MTurk respondents compared to subject pool participants [68]. Still, the findings of this study are based on self-reports and can only reflect individuals in the target age group with access to computers, tablets, or smartphones. Moreover, we also did not directly measure high-level personality variables that might indicate membership in specific classes. Recent evidence using measures of the Big Five personality factors indicates that personality is crucial to understanding why certain people conform to public health measures during the pandemic [34].

Furthermore, the data are cross-sectional and were gathered in the early stages of the pandemic. People might have changed their position with regard to public health measures and also transitioned between coping strategies as they reflected on new information as the pandemic wore on. Therefore, we cannot generalize our results beyond the early months of the pandemic. Future studies using longitudinal methods could examine the stability of coping typologies over the course of the pandemic. We also did not include cultural or political measures that also may be relevant to appraisals of the pandemic and/or compliance [69, 70]. Factors like willingness to comply with rules, conscientiousness, individualist versus collectivist values, and/or simple political affiliation might plausibly influence a person’s decision to comply or not.

### Implications

This study contributes to the psychological literature on stress and coping. We found distinct “typologies” of stress and coping, consistent with TTSC as originally conceptualized by Lazarus and Folkman [26]. The application of person-centered analyses provided a means to empirically confirm these qualitatively distinct groups of people. The study findings support one of the original claims of TTSC that people can be classified according to how they appraise and respond to stressors [25, 28]. Of course, people may show different appraisal patterns and/or different coping responses to *different* stressors. The current findings only demonstrate that, within the context of the (early) pandemic*,* there are distinct classes of people, distinguished by their appraisals and coping strategies.

This study also contributes to public health research on compliance with CDC guidelines. Previous research has not examined how perceptions about the pandemic and coping strategies predict public health compliance. Thus, the current findings illustrate the importance of psychological factors in public health research and point to the possibility of further cross-pollination between these two fields to improve public health outcomes.

## Conclusions

The present research examined typologies of stress appraisal and problem-focused coping among young adults in the context of the early stages of the COVID-19 pandemic. We found three qualitatively distinct classes of people who differed in their appraisal of the pandemic and their tendency to engage in problem-focused coping strategies. We also found that individuals who view the pandemic as central and threatening and engage in problem-focused coping were more likely than their peers to comply with guidelines recommending social distancing, mask wearing, and hand washing. These results contribute to our understanding of why people do and do not comply with public health guidelines and highlight the importance of attending to psychological variables in public health research. By understanding what drives poor compliance, we can promote greater compliance and better public health outcomes.

## Data Availability

The datasets generated and/or analysed during the current study are not publicly available because the broader research project is still ongoing and funded, but are available from the corresponding author on reasonable request.

## Abbreviations

PFC: Problem-Focused Coping
PHB: Public Health Behavior
SA: Stress Appraisal
